# Facial Emotion Recognition: A Survey and Real-World User Experiences in Mixed Reality

**DOI:** 10.3390/s18020416

**Published:** 2018-02-01

**Authors:** Dhwani Mehta, Mohammad Faridul Haque Siddiqui, Ahmad Y. Javaid

**Affiliations:** EECS Department, The University of Toledo, Toledo, OH 43606, USA; dhwani.mehta@utoledo.edu (D.M.); mohammadfaridulhaque.siddiqui@utoledo.edu (M.F.H.S.)

**Keywords:** facial expressions, emotion recognition, intelligence, augmented reality, affect, Microsoft HoloLens, human–computer interaction, sensors

## Abstract

Extensive possibilities of applications have made emotion recognition ineluctable and challenging in the field of computer science. The use of non-verbal cues such as gestures, body movement, and facial expressions convey the feeling and the feedback to the user. This discipline of Human–Computer Interaction places reliance on the algorithmic robustness and the sensitivity of the sensor to ameliorate the recognition. Sensors play a significant role in accurate detection by providing a very high-quality input, hence increasing the efficiency and the reliability of the system. Automatic recognition of human emotions would help in teaching social intelligence in the machines. This paper presents a brief study of the various approaches and the techniques of emotion recognition. The survey covers a succinct review of the databases that are considered as data sets for algorithms detecting the emotions by facial expressions. Later, mixed reality device Microsoft HoloLens (MHL) is introduced for observing emotion recognition in Augmented Reality (AR). A brief introduction of its sensors, their application in emotion recognition and some preliminary results of emotion recognition using MHL are presented. The paper then concludes by comparing results of emotion recognition by the MHL and a regular webcam.

## 1. Introduction

Humans interact socially with the help of emotions, which are considered as a universal language. These emotions surpass cultural diversities and ethnicity. Facial expressions are responsible for conveying the information, which was difficult to perceive. It gives the mental state of a person that directly relates to his intentions or the physical efforts that he must be applying for performing tasks. As a result, automatic recognition of emotion with the help of high-quality sensors is quite useful in a variety of areas such as image processing, cybersecurity, robotics, psychological studies, and virtual reality applications to name a few. Efforts in this area are being made to gather information of high-quality to meet the demands of the system so that it can read, process and simulate human emotions. Geometric and machine learning based algorithms for an effective recognition are being refined, emphasizing emotion recognition in real-time and not just ideal laboratory conditions. Hence, building a system that is capable of both face detection and emotion recognition has been a crucial area of research.

It is a well-established fact that human beings are responsible for the depiction of six basic emotions, namely happiness, anger, surprise, sadness, fear, and disgust [1]. These primary emotions form the primary classification of the study of human emotional responses. Apart from these basic emotions, several other emotions have been considered for research. These include contempt, envy, pain, drowsiness and various micro expressions. Facial expression is seen as the primary mode of recognition of human emotion. It works on facial motion and the deformations of facial features to classify them into emotion categories. This classification is based on visual information and may not be the sole indicator of emotion. Other factors also contribute to the recognition of a person’s emotional state such as voice, body language, gestures or even the direction of the gaze. Emotion recognition, therefore, demands a more precise knowledge of all these factors together with contextual information to convey more accurate results.

**Figure 1:**
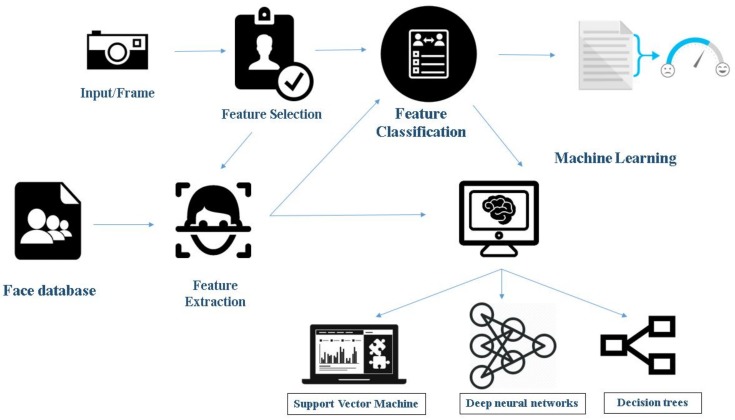
Face detection and emotion recognition using machine learning.

**Figure 2:**
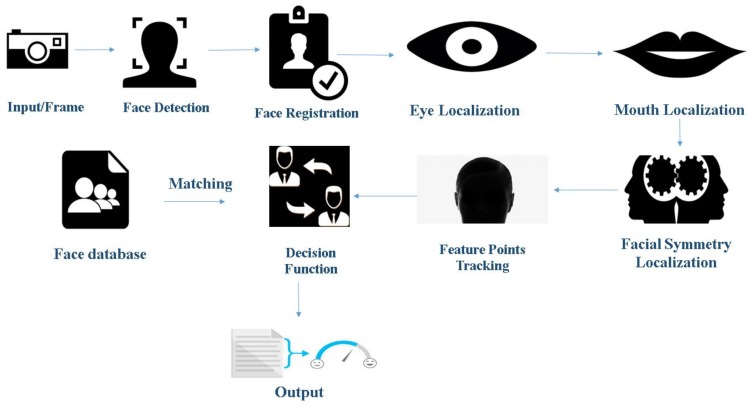
Face detection and emotion recognition using geometric feature-based process.

Facial emotional recognition is essentially pattern recognition and involves finding regularities in the set of data being analyzed. Using these regularities, faces, as well as emotions, can be recognized. Various techniques are followed to carry out the tasks that widely fall into two classes, method of parameterization, and the method of recognition. The method of parameterization includes segmentation, assigning binary labels to each pixel and detection where a boundary box is obtained when the face is located in the given data [2]. FACS (Facial Action Coding System) is an example of this method, where all facial emotions are considered and described by the contraction of facial muscles being considered as AUs (Action Units) [1,3]. These Geometric Feature-based techniques give importance to the structural shape of facial components such as nose, mouth, and eyes. The other method is appearance-based where attributes such as intensities, pixel values, and histograms are considered. After exhaustive training is done with the help of prelabeled datasets, machine learning techniques are applied to detect emotions. Figure 1 and Figure 2 give an overall idea of the use of these machine learning and geometric feature based processes, respectively, in emotion recognition. An essential requirement for the identification of the emotion by applying a machine learning algorithm is the availability of appropriate datasets for training. Different experiments have been conducted in literature with different sizes and types of data to check the maximum achievable accuracy of these algorithms. For building a robust system that recognizes basic emotions, some preprocessing is required, including image standardization, face detection, facial component detection, emotion extraction, and emotion matching or classification [4]. These tasks are becoming more challenging and complicated because of the variance in several known factors. In general, the factors that have been considered include pose and lighting conditions, gender, age, and facial hair. This study provides a brief overview of the variety of the available databases, and a comparison of the accuracy of algorithms available in the literature is made for all the described databases.

This paper presents results from several experiments, performed with the help of a popular mixed reality device known as Microsoft HoloLens (MHL) (Microsoft, Redmond, WA, USA), and a webcam for emotion recognition. The emotions considered for this experimentation include anger, neutral, happiness, sadness, and surprise [1]. As deformations of facial features are used to classify them into particular emotions, it is also important to consider the sensors of the device used for capturing the deformations in the faces. MHL provides the sensors that are essential in conducting experiments for facial emotion recognition. Depth sensors in these mixed reality devices have become very popular, allowing for the development of new algorithms for the identification of human pose, gestures, face, and facial expressions. MHL provides a variety of sensors such as an ambient light sensor, four microphones, one depth camera, an IMU (inertial measurement unit), four environment understanding cameras, mixed reality capture, and one 2.0 MP (Mega Pixels) photo/HD video camera. These sensors make the emotion recognition system robust against varying environmental conditions that may be difficult for other forms of emotion recognition systems (e.g., system having only a 2D camera). These sensors provide a high-quality input that assists in a superior dissection of subjects facial components, thus improving the efficiency of algorithms even in difficult lighting conditions.

### 1.1. Face Detection and Emotion Recognition Using Machine Learning

Facial emotion recognition [5] is a complex task and the machine learning approach to recognize faces requires several steps to perform it, as shown in Figure 1.

1. Feature selection: This stage refers to attribute selection for the training of the machine learning algorithm. The process includes the selection of predictors for construction of the learning system. It helps in improving prediction rate, efficiency, and cost-effectiveness. Many tools such as Weka and sci-kit-learn have inbuilt tools for automated feature selection.
2. Feature classification: When it comes to supervised learning algorithms, classification consists of two stages. Training and classification, where training helps in discovering which features are helpful in classification. Classification is where one comes up with new examples and, hence, assigning them to the classes that are already made through training the features.
3. Feature extraction: Machine learning requires numerical data for learning and training. During feature extraction, processing is done to transform arbitrary data, text or images, to gather the numerical data. Algorithms used in this step include principal component analysis, local binary patterns, linear discriminant analysis, independent component analysis, etc.
4. Classifiers: This is the final step in this process. Based on the inference from the features, the algorithm performs data classification. It comprises classifying the emotions into a set of predefined emotion categories or mapping to a continuous space where each point corresponds to an expressive trait. It uses various algorithms such as Support Vector Machine (SVM), Neural Networks, and Random Forest Search.

### 1.2. Face Detection and Emotion Recognition Using Geometric Feature-Based Process

The geometric feature-based approach requires several steps to perform facial emotion recognition, as shown in Figure 2 [5].

1. Image standardization: It includes various sub-processes such as the removal of noise from the image, making all the images uniform in size and conversion from RGB (Red, Green and Blue) to grayscale. This makes the image data available for image analysis.
2. Face detection: This phase involves detecting of a face in the given image data. It aims to remove all the unwanted things from the picture, such as background, and to keep only relevant information, the face, from the data. This phase employs various methodologies such as face segmentation techniques and curvature features. Some of the algorithms that are used in this step include edge detection filters such as Sobel, Prewitt, Laplacian, and Canny.
3. Facial component detection: Here, regions of interests are detected. These regions vary from eyes to nose to mouth, etc. The primary step is to localize and track a dense set of facial points. This step is necessary as it helps to minimize the errors that can arise due to the rotation or the alignment of the face.
4. Decision function: After the feature point tracking of the face using parameters such as localized feature Lucas Kanade Optical flow tracker [6], it is the decision function responsible for detecting the acquired emotion of the subject. These functions make use of classifiers such as AdaBoost and SVM for facial emotion recognition.

### 1.3. Popular Mixed Reality Device: Microsoft HoloLens (MHL)

**Figure 3:**
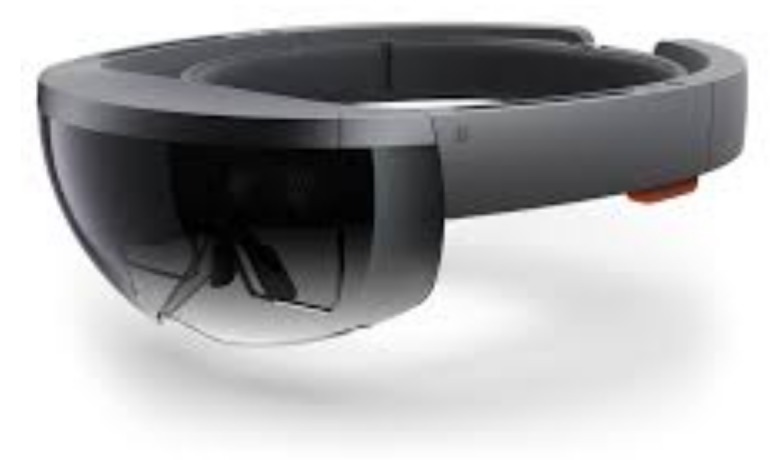
Popular mixed-reality device (MRD): Microsoft HoloLens (MHL).

In this paper, we introduce a popular mixed reality device called Microsoft HoloLens (MHL), shown in Figure 3. Mixed reality (MR) devices provide users with a combined experience of both Virtual Reality (VR) and Augmented Reality (AR). Holograms are used for interaction with the surroundings, and it helps us to create a digital world in nearby surroundings thus, forming a self-contained computer that is holographic in nature. AR is experienced when a computer generates sounds, graphics, photos, videos and displays it in 3D in our world all through the sensors. It is a blend of VR with the real world to enhance the perception of reality. In this way, the actual view of the world is modified by the computer to show different things. The only difference between VR and AR is that virtual reality substitutes the real world with a simulated holographic world and hence none of the surroundings are seen. AR is in real time and is also interactive with the environment. AR is the one, bringing out the components of the world, which is digital, into the real world as observed by the human. Hence, MHL can give us a maximum of the experiences that were available first either in only AR or only VR.

### 1.4. Sensor Importance in Mixed Reality Devices for Emotion Recognition

The slightest change in the expression is necessary to be detected to recognize the emotion portrayed by the person. Efficient and high-quality sensors are essential for measuring even an exiguous change in expression. MHL contains sensors such as an inertial measuring unit (IMU), which is responsible for keeping track of orientation, gravitational force and also the velocity of the device. The primary function of the IMU in mixed reality is to perform rotational tracking for input devices and HMDs (Head mount displays). It measures rotational movements of the yaw, pitch, and roll. For facial emotion recognition, the algorithms that use the video camera are susceptible to elements such as facial illumination environment (caused due to bad lighting or unevenly lightened scene), which affects the accuracy of the algorithm. A possible solution to this problem is to have a sensor with a scene depth information. The sensors in the depth camera use additional light source information, which, in turn, makes the capturing of the face and emotion easier in real time as they are insensitive to ambient lighting conditions. The grayscale sensing camera additionally adds up to the depth camera and hence keeps track of the heads movement, hands, and environment understanding. Other sensors like mixed reality capture help in displaying the captured emotions with ease, which is otherwise burdensome for different devices. To enhance emotion recognition of face along with other aspects such as voice, it also contains four microphones. The combination of all these sensors in this mixed reality device adds up to give a more efficient system, resulting in more accurate results, which will be shown in the experimentation section of the paper.

### 1.5. MHL Experimentation

MHL shows a merged world, that is, all the objects are merged with the places or people in the surroundings. MHL is a multi-sensor device. The sensors include one depth camera, four environment cameras, and light sensor. For human understanding, MHL has introduced spatial sound, gaze tracking, gesture input, voice support, built-in speakers, audio 3.5 mm jack volume up/down, power button, etc., which makes HoloLens more interactive and increases its usefulness. It does not require a PC connection, nor their wires in it, which makes it extremely portable and self-contained.

In this study, the importance of the sensors in the MHL is highlighted by using it to observe how the emotion recognition algorithm works in identifying human emotions more accurately. An application has been built that uses MHL to detect faces and recognize the emotion of the person in facing it. The emotions recognized are Happiness, Sadness, Anger, Surprise and Neutral. These results will be compared with the results of the facial emotion recognition experimentation, which is performed by using a simple webcam.

### 1.6. Closest Competitors of MHL

Ever since the first project called the “Google glass” came into the picture, the reality of wearable eye-wear seems to be inevitable. Since then, developers from around the world have been trying to come up with different MR devices. They all have an underlying motive to fill the users with live information, having to look at project data in a high resolution, increasing security and learning capabilities in various streams. Many such devices already on the market today are Meta, Google Glass, Meta 2, Google Daydream, Sony PlayStation VR, etc. To the best of our knowledge, the closest competitors of MHL are Google Glass and Meta 2 [7].

Google glass is an eyeglass where the lens is replaced by the optical head-up display, communicating with the internet through natural language commands. It is a smartphone in the form of glass. However, MHL is designed to recognize the vocal communication of the wearer, eye movement, hand gestures, etc. whereas Google Glass is not able to do so. Google glass was developed as a smartphone which people thought was newer but costlier, hence Microsoft HoloLens having a brighter future. The Meta 2 also feels like a complete AR experience. It offers the widest view, direct interaction with holograms and intuitive access to digital information (uses a neuroscience-driven interface). Having only a few similarities with Meta 2, MHL has proved to be better. It has features like environment understanding, human interaction with holograms, having speech control in the form of Cortana and, most important of these, being tetherless, which, as of today, is not possible with Meta 2. MHL has chosen portability over the processing power, which makes it possible to use it practically anywhere.

The structure of the paper is as follows. Section 2 gives a brief insight into techniques used for facial emotion recognition, along with a mention of the algorithms used. It also describes the types of databases that are used as data sets. Section 3 talks about our motivation to gather information and perform experiments. Section 4 describes the face detection and emotion recognition experimentation performed and the results obtained with the help of MHL and a webcam. Section 5 is a conclusion of the literature survey and the experimentation, along with which the future work is presented.

## 2. Literature Survey

In the 19th century, the worth of Emotion Recognition was recognized when “The Expression of Emotion in Man and Animals” was written by Charles Darwin [8]. This book greatly inspired the study of emotions. Emotion Recognition due to its various applications gained immense importance like a drowsy driver could be spotted using emotion recognition systems [9]. Corneanu et al., Matusugu et al., and Viola et al. [8,10,11] gave a primary classification for the emotion recognition using multimodal approaches. They primarily talked about techniques and the parameters used for emotion recognition. The methods mentioned consisted of localization of the face using detection and segmentation, which made use of Support Vector Machine (SVM) and Convolutional Neural Networks (CNN) algorithms. Along with all these techniques, Corneanu et al. concentrated on the categorization for emotion recognition considering the two principal components, one was parametrization, and the other was recognition of facial expressions. In his research, parametrization was relating the emotions detected while recognition of facial expressions was accomplished by using the algorithms such as Viola and Jones. This study also experiments with other algorithms like CNN [12] and SVM [13], and this study concludes by proving that CNN demonstrates to have comparatively better accuracy on Viola and Jones algorithms.

Matsugu et al. [10] developed the first facial ER model. This developed system claimed to be robust in appearance and independent of the subject. They used a CNN model, which was used to find local differences between neutral and emotional face. A single structure CNN was used to experiment in spite of two CNN models, which was similar to the Fasel’s model [14]. Fasel’s model had two CNNs that were independent, one was used for facial expression, and the other was used for face identity recognition. Furthermore, an MLP was used to combine them. The experiment was performed with images of various types and achieved a performance rate of 97.6% for 5600 still images of 10 subjects. Tanaya et al. [15,16] applied a Curvelet-based feature extraction. Here, they took advantage of the discontinuities in 2D functions, which were represented by Curvelet. In their work, they converted the images to grayscale. These images were then exploited to the 256 resolution further to 16 and then to 8 and 4 resolution, respectively. Later in his work, curvelet was used for training the algorithm. The reason to follow this flow was that the person image would be recognized by bigger curves, which are present at the lower bit resolution if initially a person’s face is not recognized in the 8-bit image. Finally, the One-Against-All (OAA) SVM method was performed, and the results of wavelet and curvelet-based methods were compared on various known databases in which curvelet method proved to have a higher performance than wavelet methods.

Li et al. [17] mentioned that recognition of emotion is completely based on visual information. He conducted an experiment that was based on the recognition of smiles. The subjects were made to depict the smiles. The comparison was made between 3D and 2D emotion recognition. To complete the test, symmetric property of the face method was used for registration. The cubic spline interpolation method was used to overcome holes in the image which were caused due to dark hair on the face. Feature extraction was done by using Principal Component Analysis (PCA); along with that, a LIBSVM (library for SVM) package was used in accordance with a linear discriminant classifier to execute SVM for emotion recognition. Linear Discriminant Analysis (LDA) and SVM, and both had a performance rate of more than 90% for 3D images and also, when considered for 2D images, it had performance rate around 80%.

Anil et al. [4] made a succinct survey of the techniques, which were used for emotion recognition along with the accuracies measured on various databases. A brief comparison was made between the 2D and 3D techniques. The standard classification was done which consisted of algorithms falling into the category of Geometric Feature-based and Appearance-based methods [18]. The methods observed were Gabor Filters [19] , Local Directional Number Pattern [20], Patched Geodesic Texture [15], Curvelet Feature extraction [4], FARO: Face Recognition Against Occlusions and Expression Variations [21], and Gradient Feature Matching, which was observed for Expression-Invariant Face Recognition with the help of a Single Reference Image [22]. In this approach, both local and host features were taken into account to achieve a higher rate of performance. The few databases used in this study were CMU Advanced Multimedia Processing (AMP) database, AR database, Cohn–Kanade (CK) database [23], Yale database, Japanese Female Facial Expression (JAFFE) database [24], and /hlBinghamton University 3D Facial Expression (BU-3DFE) database [25,26]. His work concluded that even in the case of occlusions the Bag of Words and FARO methods could recognize the emotion. After the Anil et al. work was finished, Matthews and Baker [27] extended the work by mentioning there were two types of (Active Appearance Model) AAM, Independent AAMs—they perform linear modeling and also the appearance of deformable objects (both separately), while, for the combined AAMs, they use a single set of parameters for both shape and appearance.

Mohammed et al. [19] introduced a new algorithm that was combined using both Bilateral Two-dimensional Principle Component Analysis (B2DPCA) and an extreme learning machine (ELM). Along with these two, a curvelet-based algorithm was also used. Significant experimentation was performed on databases like FERET [28], Faces94 [29], JAFFE [24], Georgia Tech [30], Sheffield [31], ORL [32], and YALE [33]. The curvelet features were used as input to the ELM after being dimensionally reduced with the help of B2DPCA for learning the enormous model and hence emotion recognition was achieved at a very high rate. This method was independent of any hidden neurons plus the training datasets size.

Rivera et al. [20] talked about Local Directional Number Pattern (LDN), a method that had the capability of outperforming the code more discriminately than many other existing methods. Using a compass mask, computation of the structure of each micropattern, which was responsible for extracting the directional information, was achieved. They also used prominent direction indices along with the signs that help in distinguishing the similar structural patterns having different intensities. These methods were tested under various conditions like noise, timelapse, and illumination. The accuracies of that computed LDN with different expressions were observed and compared. The use of varying compass masks (a derivative-Gaussian and Kirsch) was analyzed by them to extract information like direction and their performance rates on multiple applications. In his study, he suggested that LDN for face and expression recognition approach is very robust and reliable under different lighting conditions and could recognize even subtle emotions.

Kahou et al. [34] worked on a hybrid CNN—RNN (Recurrent Neural Network) architecture for emotion recognition. The hybrid approach was done by performing aggregation of CNN and RNN. The paper explored three CNN structures:

- a very deep one with 3×3 frame size;
- a three-layer with 5×5 filter size; and
- finally in the third one increased the filter size to 9×9.

The work done by them used RNN to aggregate the frame features. The main reason to do this was that RNN could learn from an event irrespective of the time at which it must have occurred in a sequence. They performed a feature level and a decision level fusion, which, in turn, provided a significant improvement. The fusion with feature level was done using an MLP that had different hidden layers for each modality. In the fusion of decision level, the weighted sum of the class probabilities that were estimated was used. This architecture outperformed all the other methods like the aggregation of CNN-RNN performed and averaged of per frame based classifications.

Enrique Correa et al. [35] worked on three architectures of the deep neural network that were used for emotion recognition, and, out of those, the best one was chosen for further optimization.

- Their first architecture was based on Krizhevsky and Hinton [36]; it consisted of three convolutional layers with two fully connected layers. The process had reduced size of images through max-pooling and also, to overcome overfitting, it had a dropout layer.
- In the second architecture, instead of two fully connected layers, they applied three fully connected layers, with local normalization to speed up the process.
- The third architecture had three different layers like one convolution layer, one local contrast normalization, and max-pooling layer, and, in later stages, they added the third max-pooling layer to reduce the number of parameters.

The accuracies achieved were 63%, 53%, and 63%, respectively, for the above three architectures. The paper observed that having a smaller network size reduces the performance of the original network more than expected. Hence, the second architecture was not competitive to the remaining two architectures. Yu et al. [37] proposed a technique where the face detection had three state-of-the-art face detectors followed by multiple (seven hidden layers) deep CNN models. There, the CNN were initialized randomly and retrained on a larger dataset. For combining the CNN, two methods were used: minimizing the hinge loss and minimizing the log-likelihood loss. The experiments were done using Static Facial Expressions in the Wild (SFEW) database [38], SVM was trained and tested on the output responses of the concatenated network. The results are shown in Table 1. Shan et al. [39] indicated that Local Binary Pattern (LBP) features could be derived at a faster rate as compared to the Gabor wavelets. The discriminative features were stored in small representations, and it learned more LBP features with AdaBoost (Adaptive Boosting). Along with these stored discriminative features, it was proven to give the best recognition to SVM. The study performed algorithms other than SVM like linear discriminant analysis and template matching. Zang et al. observed improvement by the work done by (LBP feature) [39,40], and it also performed better than Gabor filtering emotion recognition. It used AU intensity detector, which was regression-based, emotion clustering for recognition of emotions and also unsupervised facial point detector. The facial detector outperformed AAM and Constrained Local Model (CLM) by 13% and 9%. The summary of the literature survey is presented in the form of Table 1. It gives a brief idea of the accuracies achieved through different methods and use of databases. The table consists of the technique used, the database used, accuracies achieved and the drawbacks.

### 2.1. Database Description

Databases form an essential part of the training of machine learning algorithms. Approaches using supervised learning for emotion classification require a vast and varied dataset for proper training and testing, hence using proper training and testing datasets are of great importance. The correctness of the results is remarkably affected if the database is in abundance and is highly accurate. Even though there are many available databases of facial images for recognition of face [41], a lesser number of databases have been formed, which support recognition of facial emotions. The type of images in most of the databases offered are 2D. However, some offer 3D images captured through stereography or 3D scanners. 3D images efficiently improve recognition accuracy even in unevenly lighted conditions. These various classes of databases are being used for training human facial expressions for recognizing emotions.

The images/video recordings in the databases can be divided into two major categories: spontaneous and posed datasets. Each of them has advantages and disadvantages.

- Posed Datasets: Popular for capturing extreme emotions. The disadvantage is the artificial human behavior.
- Spontaneous Datasets: Natural human behavior. However, it is extremely time-consuming for capturing the right emotion.

Thus, we can conclude that a database for facial emotion recognition should have a combination of both the categories. A close relationship exists between the advancement of face recognition algorithms and the availability of face databases. This section describes in depth about the various RGB databases, the thermal image databases, and the 3D image databases with their characteristics.

#### 2.1.1. RGB Databases

Many databases are available online for public use for conducting experiments on facial emotion recognition. However, only a few of them contain 3D information. The very first comprehensive database to get public was the Cohn-Kanade (CK) [23] database. The CK database was formed in a sequence, and each sequence was labeled by the desired emotion to be expressed and as observed by the facial movements using FACS [1,3]. However, the major drawback of this system was that the sequences were not verified against the real facial emotions they contained. The emotion label referred to what expression was requested rather than what could have been performed. The CK database had only frontal views of the subjects, and it also did not have a variety of data available, namely, a few gender sets and age. In the CK database, it had similar illumination ranges. The biggest drawback of this RBG dataset is the lack of intensity labels. Hence, modifications were done, and, furthermore, this database was extended to CK+ [53]. In the later stages, CK+ had increased their number of samples and also had spontaneous expressions of the clicked images of subjects. There was an addition of 593 (327 sequences having discrete emotion labels) sequences with more number of frames per sequence. CK+ provided FACS coding with the validated emotion labels. CK+ had posed as well as spontaneous facial emotions. CK+ database has a frame resolution of 640×490.

JAFFE [24] is a database of static images that was created in laboratory conditions where emotions were acted. The database was formed by 213 images having seven facial expressions, which were enacted by 10 Japanese female models. It has a resolution frame rate of 256 × 256 with facial expressions labels. It has only posed expressions. The data sets improved their quality when the MMI (named after their creators **M**aja Pantic, **M**ichel Valstar and **I**oannis Patras) [54] datasets were created. The MMI datasets consisted of many of the AU expressions of the FACS. It had 43 subjects (1280 videos and over 250 images) with a resolution frame rate of 720 × 526. The MMI database was formed with AU label for the image frame with apex facial expression in each image sequence. It had both posed and spontaneous facial expressions. In the RGB databases, JAFFE was all gray, and MMI was colored. The MULTI PIE [55] database had multiple views of subjects at various angles, also adding a variety of illumination situations. It was made up of more than 750,000 images recorded in up to four sessions over the span of five months by considering 337 people as subjects plus high-resolution frontal view images as well. These 337 subjects were considered under 19 illumination conditions while being imaged, for displaying a range of facial expressions, and they were also imaged for 15 viewpoints. In total, the database contains more than 305 GB of face data.The AFEW [56] database has in total six primary emotions with the special mention of age, pose and gender. In comparison to AFEW, SEMAINE [57] had labels of shakes and nods, laughs, states while interacting with agents. The SEMAINE database consists of 150 participants with a total of 959 conversations, which were conducted with individual Sensitive Artificial Listener (SAL) agents that lasted approximately five minutes each. SEMAINE had information of the FACS, like laughter identification and annotation on selected extracts.

#### 2.1.2. Thermal Databases

Presently, thermal databases are used for a variety of reasons like for target acquisition in the military, medical diagnosis, etc. Thermal IR sensors are the ones who are responsible for capturing the emitted heat patterns by the objects. Hence, thermal Infra-Red (IR) imagery is not dependent on ambient lighting conditions, thus forming a promising alternative for recognition of facial emotions. The high use of the thermal database is due to the reason that they already contain the data in the RGB form. Thermal databases, however, are very few, and primarily they lack thermal information. One of the drawbacks is that the database includes only posed facial images. The very first ones only had three expressions (laugh, surprise and angry) with different poses of the head in different illuminations. The very first ones are called the Imaging, Robotics and Intelligent System Database (IRIS) [58] and National Institute of Standards and Technology (NIST) [59] databases. The IRIS database consisted of 30 individuals and their images with a resolution of 320 × 240 in which 28 were men, and two were women. Two different sensors were used to form this database, the first is Thermal—the Raytheon Palm-IR-Pro (Raytheon, Waltham, Massachusetts, USA)—and the second is Visible—the Panasonic WV-CP234 (Panasonic, Kadoma, Osaka Prefecture, Japan ). A total of 11 images per rotation was recorded where 176–250 images per person were recorded. Out of 3058 images, there are in total 1529 images that are thermal. NIST consists of 1573 individual images that have 78 female images and remaining male images. It consists of side and front profiles.

The Natural Visible and Infrared Facial Expression (NVIE) [60] database gives a collection that consists of six expressions in total. They are both in posed and spontaneous expressions, while posed expressions are both with and without glasses. It has more than 100 subjects; the recording was done simultaneously by a thermal infrared camera and visible camera. It had illumination from three different positions. The drawbacks of this database were that not all of the six expressions recorded are spontaneous. One more disadvantage is the gap between video clippings is 1–2 min long, which is too short for a person to establish a neutral status. The Kotani Thermal Facial Emotion (KTFE) [61] database, like NVIE databases, have all six spontaneous and triggered emotions. This database contains 26 subjects who are Vietnamese, Japanese, and Thai from 11-year-olds to 32-year-olds with seven emotions. It has 131 GB of visible and thermal facial emotion videos.

#### 2.1.3. 3D Databases

It is difficult to handle subtle facial expression changes and large pose variations in 2D image-based databases. 3D facial expression databases hence come into the picture to facilitate the recognition of subtle emotions to improve the overall accuracy of algorithms. One of the most used 3D databases for emotion recognition is the BU-3DFE [25] database. The database was made of 100 subjects in which 56% were female, and 44% were male with 2500 facial expression models. Due to its variety of subjects like Indian, Latino, White, Middle-East Asian, it was considered a well-formed database with a variety of ethnic/racial ancestries. Six different expressions were depicted using four different levels of intensity. The database is high resolution and has videos from 101 subjects that were taken, each consisting of more than a hundred frames. The Bosphorus database consists of many expressions at the low-cost ethnic diversity of subjects in comparison to BU-3DFE [62]. The Bosphorus database has systematically varied poses with different types of occlusions. It has some unique properties that include a rich variation of head pose, face occlusion, and facial expressions are composed of a judiciously selected subset of Action Units. However, both Bosphorus and BU-3DFE have posed expressions.

## 3. Motivation

It was only in the 1990s that the term Augmented Reality (AR) came into existence, following which was the Virtual Reality (VR), which was not patented until 1962. These terms are now gaining importance as the market has started developing devices based on AR and VR. However, not much work has been done with these devices in real-time. Research is underway on use of these devices for various applications including judging a suspicion by the military, helping a medical student learn surgery, emotional branding to create an emotional connection with customers, and observing behavior in Congress during discussion of critical issues. Automated verification of human identity is indispensable in security and surveillance applications these days. Biometric authentication schemes based on video modalities are non-intrusive and are therefore more suitable in real-world settings compared to the intrusive methods like fingerprint and retina scans. This forms the basis of the research for observing and detecting emotions through facial expressions in AR. Facial expressions constitute the prime source of emotional recognition supported in some cases by various other modalities such as speech or physiological signals. A high accuracy algorithm supported by the exemplary sensors (providing high-grade input data) can achieve useful results that can be used to recognize the emotions in real time in AR. With the whole new world of mixed reality devices gearing up to astonish the world with its multiple uses, testing the algorithms for emotion recognition with such devices plays a crucial part in research for both the developers and users. For this study, the mixed reality device introduced by Microsoft, the Microsoft HoloLens (MHL), has been used.

MHL is more than just Augmented Reality (AR) and Virtual Reality (VR); it is all about Mixed Reality. Microsoft HoloLens has introduced a new type of computing called the Holographic computing, which is developed with high-quality sensors. Microsoft is inviting people to build applications for the HoloLens in the field of 3D, medical purposes, and STEM learning (STEM education is an interdisciplinary approach to learning where rigorous academic concepts are coupled with real-world lessons as students apply science, technology, engineering, and mathematics in contexts that make connections between school, community, and work.) The use of a mixed reality device for emotion recognition will get one’s feet wet in practical purposes like cyber-security, learning the mental state of a person, etc. The emotion detection can assist to find the concealed emotions when a person is being sarcastic at times or happy or sad or is having some suspicious intentions. MHL offers one of the most influential factors for experimenting with such MR devices, the choicest sensors. High-quality, different sensors allow the development of new systems for the recognition of human pose, gestures, face, and facial expressions. With the help of sensors, the existing algorithms can be tweaked based on, e.g., on an RGB camera and hence improved recognition efficiency in difficult illumination conditions along with better localization of facial parts. MHL has many high-quality sensors, which include one depth camera, four environment cameras, and light sensors. For human understanding, MHL has introduced spatial sound, gaze tracking, gesture input, voice support, built-in speakers, audio 3.5 mm jack and volume up/down, power button, etc., which makes HoloLens more interactive and increases its usefulness. Making facial emotion recognition work with Microsoft HoloLens has more effective results as only the observer has to wear the MHL, and it has no amount of sensors put on the subject’s body. An experiment was performed with this MR device and a webcam to compare the achieved accuracy.

## 4. Experimental Results

This section presents an experiment with the help of MHL and a webcam. A brief comparison is done, which is supported by results.

### 4.1. Experimental Setup

The setup for this experiment included Microsoft HoloLens, a webcam and a laptop/desktop with Windows 10 version (Microsoft, Redmond, WA, USA). The standard webcam was used along with the desktop or laptop while the Microsoft HoloLens has its own Windows 10 operating system. The webcam used had a single camera while the MHL uses six individual cameras to capture images from various angles and then combines them [63]. Due to this merging, the best exposures of each image sections make MHL pictures better in quality. Three people participated, as the subject for this experiment to detect five different emotions with no change in the lighting. In addition to those 15 images taken by MHL and 15 images taken by a webcam, a dataset of 200 images (100 taken by MHL and 100 taken by webcam) was included to provide concrete comparison results for the experiment. The experiment was performed under normal laboratory conditions, without any background being tampered with. All light sources that maintained ambient lighting in the laboratory were switched on while conducting the experiment. We also captured the emotions from various angles and with different environments in the lab, in order to reduce the effect of rotation in-variance and background. Likewise, some emotions were captured with the subject standing, and some were captured while sitting on a chair. Emphasis was given to capturing poses as natural as possible in a typical environment.

### 4.2. MHL Results

The experiment focused on detecting the emotions of the subjects under consideration. The first step of the process was to detect the face and then the facial expression of the person. The analysis was done using the existing Microsoft Application Programming Interface (API) titled “Emotion API” [64]. It took the input image and set up a bounding box across the face in the image. The API used Microsoft’s cutting-edge cloud-based emotion recognition algorithm. As three different subjects enacted the emotions, the experimenter who is wearing the Microsoft HoloLens was taking the video. The process was done in the Microsoft HoloLens and the emotion depicted was displayed. The five emotions taken into consideration for this experiment are happiness, sadness, anger, surprise and neutral. The final result images displayed attributes like gender, age, glasses, beard, and emotion. Unity (Unity Technologies, San Francisco, USA) Visual Studio Community 2015 was the platform used for this experiment.

### 4.3. Webcam Results

Using a webcam, a similar experiment with the same three subjects was performed and compared. An existing API was used for comparison. The API took an input image as well as a box detecting the face. These emotions are communicated cross-culturally and universally via the same basic facial expressions, where they are identified by Emotion API [64]. The API used Microsoft’s cutting-edge cloud-based emotion recognition algorithm. Using the webcam, a picture of the subject depicting the expression was taken and then processed. Here, for the sake of comparison, same attributes of all the subjects were considered, i.e., the values of expressions like happiness, sadness, etc.

### 4.4. Analysis

The MHL and a webcam were used to detect emotions with a Microsoft emotion API. The results are shown in Table 2 where the comparison is shown in the form of which emotions were identified for the same environmental conditions. The result from the standard webcam is tabulated in the form of probability of the emotion, where one will be the absolute surety for a particular emotion. The results were displayed for all the five emotions, and the one with the highest probability was taken as the observed emotion. There were a few more emotions (such as disgust and contempt) that were supported by the API but were not considered for this study because they were too hard to enact. In a few cases, the probability was very high, and the emotions that were detected showed high accuracy. This can be observed in the first image of the Table 2 where the emotion happy is detected with a probability of 0.99999. In certain cases, the probability of an emotion identified was confusing and was not at all near to 1; however, it had a considerate amount of accuracy (>0.5), so that it could be concluded that the emotion depicted falls into which category. For instance, image 15 of Table 2 shows that the emotion was not recognized very accurately, but, among the detected emotions, the probability for emotion angry is sufficiently higher than other emotions (0.575), and hence the emotion angry can be inferred from that.

In the case of HoloLens, the emotions depicted were recognized with remarkable accuracy. In this case, the result was not in the form of probabilities of the detected emotions. Instead, there was one pure emotion that was detected. It was also able to identify specific other features such as gender, age, mustache, beard, etc. of the subjects. The square boxes seen in the Table 2 of MHL section are the ones recognizing the faces of subjects. Although some of the recognitions were not that accurate, such as the age, a few of them were determined somewhat accurately such as gender, mustache, etc. When compared with the standard webcam, it can be observed that the recognized emotions of the subjects from MHL were more accurate than the webcam. It could be seen in Table 2, even in the case of subtle emotions and at a low intensity of emotions, that most of the times MHL detected the emotions more accurately than a webcam. This can be seen in images 13 and 14 of Table 2. The factors affecting this accuracy can be subjected to the more advanced camera in the HoloLens with more number of sensors that are inbuilt.

**Figure 4:**
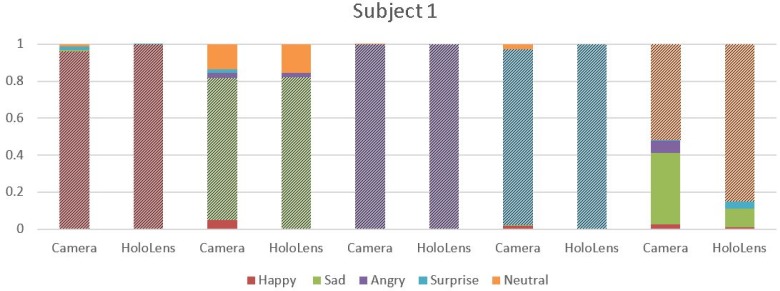
Comparison of average emotion recognition accuracy for the five emotions for Subject 1 using MHL and webcam.

**Figure 5:**
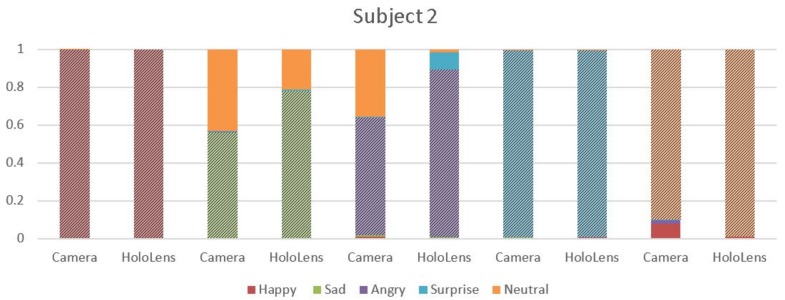
Comparison of average emotion recognition accuracy for the five emotions for Subject 2 using MHL and webcam.

**Figure 6:**
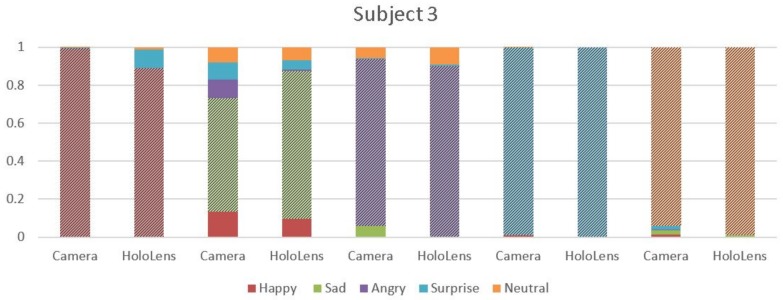
Comparison of average emotion recognition accuracy for the five emotions for Subject 3 using MHL and webcam.

Graphical representation of the results is reported in the shown bar graphs in Figure 4, Figure 5 and Figure 6, where the highlighted region depict the emotion recognized. The *x*-axis represents the emotions recognized through camera/MHL, and the *y*-axis represents the accuracy achieved, 0 indicating the absence of emotion and 1 indicating emotions with an absolute surety. In Figure 4, considering the emotion “Happy” with red color, it is seen that MHL has higher accuracy and it does not mix the emotion with the other emotions, unlike the webcam. The camera, despite having accuracy higher than 0.9, it still confuses it with emotions like sad, surprise and neutral. For the emotion “Sad”, which is green in color, comparing the results of MHL and camera, it is observed that MHL has a lower tendency to misread an emotion. While MHL confuses the sad emotion with emotions like neutral and angry, the camera misreads it for a greater number of emotions like happy, neutral, angry and surprise. Emotions “Angry” and “Surprise” have almost the same accuracy with only one emotion misread by the camera for emotion “angry” and two emotions for “surprise” and no emotion misread by the MHL. The emotion in orange is for neutral. The MHL has a higher accuracy for neutral while the webcam is confusing it with the sad emotion. The bar graphs evidently show that the accuracy is significantly affected by the use of sensors in the mixed reality device, which is having higher accuracy for the predicted emotions and a lesser number of misread emotions.

**Figure 7:**
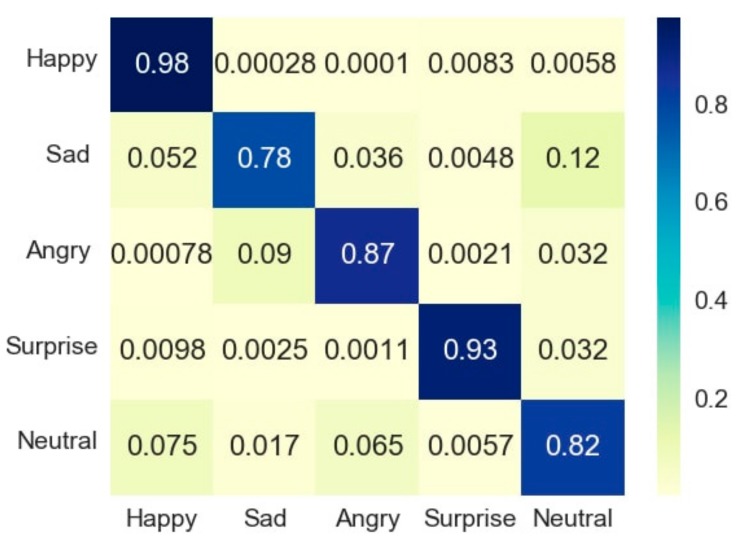
Confusion matrix of webcam-based emotion recognition results for the complete dataset.

**Figure 8:**
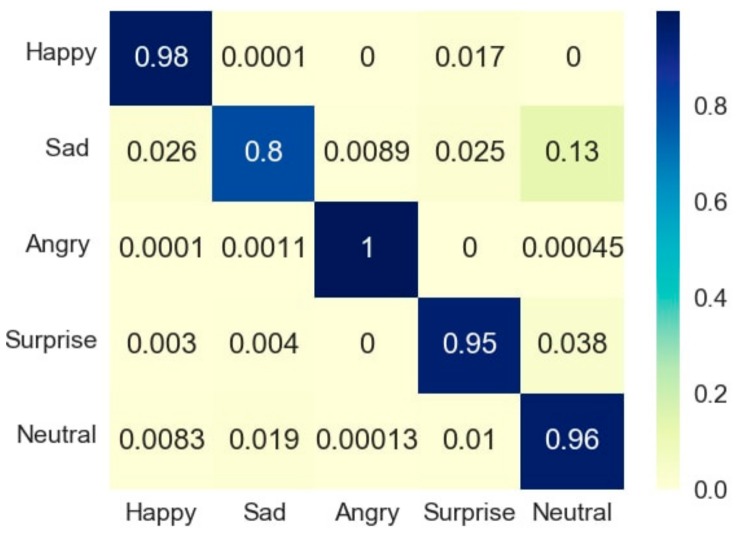
Confusion matrix of MHL-based emotion recognition results for the complete dataset.

The confusion matrices are the average of the results obtained from the whole dataset of 200 images and 20 images per emotion per device. Figure 7 and Figure 8 show the confusion matrices (in the form of heat maps) of the whole dataset (100 images taken by MHL (100 images, 20 images of each of the five emotions) and 100 by a webcam (100 images, 20 images of each of the five emotions). Figure 7 and Figure 8 show that the measured emotion through a dataset of 200 images remained in alignment with the results provided by the images of the three subjects.

### 4.5. Limitations

After conducting the experiment, some of the observed limitations are listed below:

1. MHL was made to detect the emotions enacted by the people. As people found it difficult to hold the emotions that were depicted, the accuracy of the algorithm was affected.
2. People had to give several shots for detection of the ‘SAD’ emotions, as detection of the ‘SAD’ emotion was a major limitation of MHL.
3. MHL code runs in video mode and not the real-time mode, due to which, for every real-time change in emotion, the MHL has to be set up again to detect it.
4. Technical support was not very available for MHL since a limited amount of work is done using it.
5. Expressions of people are changing every minute, rather every mini-second, as a result of which it is challenging for MHL to work with detection of face and recognition of emotions in real time.

## 5. Conclusions

The face detection and emotion recognition are very challenging problems. They require a heavy effort for enhancing the performance measure of face detection and emotion recognition. This area of emotion recognition is gaining attention owing to its applications in various domains such as gaming, software engineering, and education. This paper presented a detailed survey of the various approaches and techniques implemented for those approaches to detect human facial expressions for identification of emotions. Furthermore, a brief discussion on the combination of steps involved in a machine learning based approach and geometric-based approach for face detection and emotion recognition along with classification were described. While reporting this survey, a comparison of accuracies was made for the databases that were used as datasets for training and testing as shown in Table 1. Different kinds of databases were described in detail to give a brief outline of how the datasets were made, whether they were posed or spontaneous, static or dynamic, experimented in a lab or non-lab conditions and of how diverse the datasets are. A conclusion derived from this survey of databases is that RGB databases lack the intensity labels, making it less convenient for the experiments to be performed and, hence, compromises on the efficiency. The drawbacks of thermal databases are that it does not work with pose variation, variation in temperature, aging and different scaling (e.g., identical twin problem). Disguises cannot be captured if the person has put on glasses. Thermal images have a very low resolution, which affects the database quality. The 3D databases are not available in abundance to perform experiments and improve accuracy. The accuracies of different algorithms with these databases were also mentioned, which showed that there is scope for improvement in the field of emotion recognition regarding accuracy and for detecting subtle micro-expressions.

Later, a quick comparison was made between MHL and existing AR devices. An experiment was further performed using two different devices: the Microsoft HoloLens and a webcam. Five expressions—anger, neutral, happy, surprise and sad—were depicted by three subjects selected. The primary limiting factor affecting the accuracy of both the devices was that the emotions to be predicted had to be posed for a longer time. As it was difficult to hold on to the sad emotion until its detection, it created a serious issue. The experiment was conducted in a lab with different light conditions, and it was proven through this experiment that, because of the sensors available in MHL, it gives better accuracy in emotion recognition than the webcam with similar conditions. Through this experiment, we derived that sensors play a very influential role in recognizing an emotion. A number of high-quality sensors are required for capturing subjects emotions in real time with better accuracy in different environmental conditions. The results achieved by this experimentation are presented in the form of a comparison table that contains the predictions and the probability of the emotions. The left column of the table is for camera deductions where all the emotions with their values are seen, and the range with a maximum value of emotion, considered in the right column, is for Microsoft HoloLens, where gender, age, and emotion of the subject are mentioned. The paper concludes by suggesting limitations of the device and the problems that were observed while experimenting.

Mixed Reality is the future. The device Microsoft HoloLens available can be used to improve the accuracy of the current experiment by exploiting the use of its high-quality sensors, and more robust testing can be done using an extensive database for real-time emotion recognition. Expression change should be detected as soon as a person’s expression changes with time. This brings us to a conclusion that we will be making the algorithm work in real time. Secondly, the accuracies of the recognition of emotions, notably the “Sad” emotion, will be improved. Thirdly, various algorithms will be used for testing and validation, in order to see which one of these algorithms have the best accuracy in recognizing the emotions. In addition, the bug observed during experimentation in the previous version of MHL is now removed, and hence experiments can currently be performed multiple times quickly.

## Figures and Tables

**Table 1:** Comparison of the accuracies achieved through various techniques.

Author Name	Technique Used	Database Used	Emotion Recognition Accuracy	Emotions Considered	Drawbacks
Mutsugu [10]	Convolution neural network (CNN) Curveletface with LDA Curveletface with PCA	Still images own database (5600 images)	97.6%—CNN 83.5%—Curveletface + LDA 83.9%—Curveletface + PCA	Happy, Neutral and Talking	System insensitive to individuality of facial expressions mainly by virtue of the rule-based facial analysis
Zhang [42]	Patched based 3D Gabor filters SVM and Adaboost	JAFFE C-K	92.93%—JAFFE 94.48%—C-K	Happy, Neutral, Sadness, Surprise and Anger, Fear and Disgust	JAFFE DB requires larger sizes of patches than the CK DB to keep useful information
Hayat [43]	SVM with clustering algorithms	BU 4DFE	94.34%	Anger, Disgust, Happiness, Fear, Sadness, and Surprise	——
Hablani [44]	Local binary patterns	JAFFE	Person dependent— 94.44% Person independent— 73.6%	Happy, Neutral, Sadness, Surprise and Anger, Fear and Disgust	Manual detection of face and its components, experimented only on JAFFE DB
Zisheng [45]	PHOG (Pyramid Histogram of Oriented Histogram) descriptors	C-K	96.33 %	Happy, Neutral, Sadness, Surprise and Anger, Fear and Disgust	——
Lee [46]	Sparse Representation	JAFFE BU 3DFE	Person dependent - 94.70%—JAFFE Person independent - 90.47%—JAFFE 87.85%—BU 3DFE	Happiness, Disgust, Angry, Surprise, and Sadness	The face images used in the experiment were cropped manually
Zheng [47]	Group sparse reduced-rank regression (GSRRR) + ALM	BU 3DFE	66.0%	Happiness, Fear, Angry, Surprise and Sadness	Implementation of new method with less accuracy
Yu [37]	Deep CNN, 7 hidden layers with minimization of hinge loss	SFEW	61.29%	Happiness, Disgust, Fear, Angry, Surprise and Sadness	Less accuracy through more networks
Dornaika [48]	PCA + LDA	CMU	Above 90%	Happy, Neutral and Disgust	No non-linear dimensionality reduction techniques (kernel- and manifold-based methods) for facial expression representation, which are known for an increased discrimination
Meguid [49]	Random forest classifiers	AFEW JAFFE-CK CK-CK	44.53%—AFEW 54.05%—JAFFE - CK 90.26%—CK - CK	Happiness, Disgust, Fear, Angry, Surprise and Sadness	Assumes that the progression from one “universal” expression (the source) to another (the sink) involves a sequence of intermediate expressions pertaining to the former or the latter. In truth, these intermediate expressions may contain elements of “non-universal” expressions
Zhang [40]	SVR based AU intensity	C-K	90.38%	Happiness, Angry, Surprise and Sadness.	——
Zhang [50]	NN based Facial emotion recogniser	C-K	75.83%	Happiness, Disgust, Fear, Angry, Surprise and Sadness	Weak affect indicator embedded in semantic analysis and emotional facial expressions to draw reliable interpretation
Wu [51]	Gabor motion energy filters	C-K	78.6%	Happiness, Angry, Surprise and Sadness.	Low accuracy
Jain [52]	Latent-Dynamic Conditional Random Fields (LDCRFs)	C-K	85.84%	Happiness, Disgust, Fear, Angry, Surprise and Sadness	——
Shan [39]	Local Binary Patterns, SVM, Adaboost LDA	C-K	89.14%	Happiness, Disgust, Fear, Angry, Surprise and Sadness	Recognition performed using static images without exploiting temporal behaviors of facial expressions
Li [17]	PCA, LDA and SVM	29 Subjects	3D Database—Above 90% 2D Database—Above 80%	Happiness, Sadness, Neutral, and Anger	Small sized-database used
Mohammed and Mandal [15,19]	Patched geodesic texture technique curvelet feature extraction, gradient feature matching	JAFFE BU—3DFE	Angry—90%—JAFFE Disgust emotion—78%—JAFFE 99.52%—BU—3DFE	Happiness, Disgust, Fear, Angry, Surprise and Sadness	Consideration of few emotions
Rivera [20]	local directional number pattern	29 Subjects	92.9%	Happiness, Sadness, Neutral, and Anger	Very small number of databases used

**Table 2:** Comparison of emotion recognition using the HoloLens and a webcam.

Emotion Recognition with Camera	Emotion Recognition with Hololens	Emotion Recognition with Camera	Emotion Recognition with Hololens
1 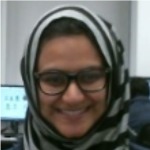 Happiness:9.9996x10${}^{-1}$ Sadness:1.0551x10${}^{-8}$ Surprise: 2.33121x10${}^{-8}$ Anger: 1.822721x10${}^{-7}$ Neutral: 1.153463x10${}^{-7}$	2 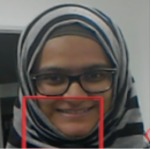 Gender: Female Age: 22.8 Emotion: Happiness Sadness: 0 Surprise: 0.001 Anger: 0 Neutral: 0	3 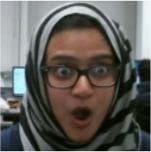 Happiness:1.745x10${}^{-10}$ Sadness: 2.9231x10${}^{-11}$ Surprise: 9.999x10${}^{-1}$ Anger: 2.807458x10${}^{-6}$ Neutral: 4.457893x10${}^{-9}$	4 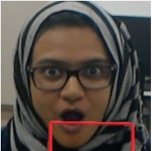 Gender: Female Age: 23.6 Emotion:Surprise Happiness:0.001 Sadness: 0 Anger: 0 Neutral: 0
5 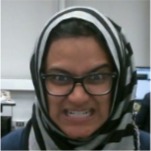 Happiness: 2.219x10${}^{-3}$ Sadness: 8.719054x10${}^{-6}$ Surprise: 2.328x10${}^{-5}$ Anger:9.962646x10${}^{-1}$ Neutral: 6.154489x10${}^{-5}$	6 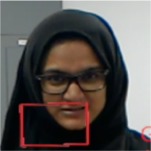 Gender: Male Age: 31.2 Emotion:Anger Happiness:0 Sadness:0.001 Surprise:0 Neutral:0	7 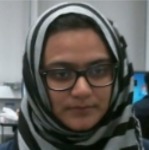 Happiness:9.9996x10${}^{-1}$ Sadness:1.0551x10${}^{-8}$ Surprise:2.33121x10${}^{-8}$ Anger:1.822721x10${}^{-7}$ Neutral:1.153463x10${}^{-7}$	8 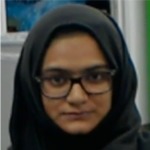 Gender: Male Age: 28.1 Emotion:Neutral Happiness:0.009 Sadness:0.1 Surprise:0.001 Anger:0.04
9 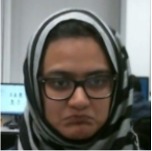 Happiness:5.2813x10${}^{-5}$ Sadness:0.7741635 Surprise:0.0001599687 Anger:0.02538101 Neutral:0.02138592	10 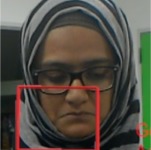 Gender: Female Age: 24.9 Emotion:Sadness Happiness:0 Anger: 0.022 Surprise:0 Neutral:0.156	11 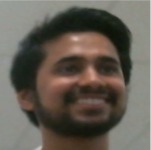 Happiness:0.999968 Sadness:4.31569x10${}^{-9}$ Surprise:3.5701x10${}^{-7}$ Anger:2.009572x10${}^{-9}$ Neutral:28527x10${}^{-6}$	12 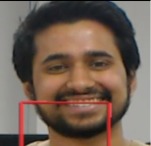 Gender: Male Age: 36.1 Emotion: Happiness Sadness:0.0001 Surprise:0.1 Anger:0 Neutral:0.0099
13 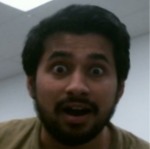 Happiness:4.10x10${}^{-5}$ Sadness:3.93907x10${}^{-7}$ Surprise:0.9976828 Anger:3.272228x10${}^{-5}$ Neutral:0.00010395	14 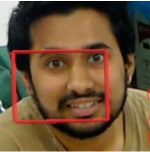 Gender: Male Age: 32.2 Emotion:Surprise Happiness:0 Sadness:0 Anger:0 Neutral:0	15 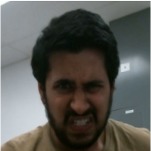 Happiness:0.00316 Sadness:0.05386358 Surprise:0.0040604 Anger:0.5759627 Neutral:0.05620338	16 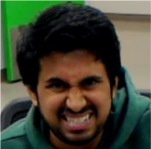 Gender: Male Age: 36.9 Emotion:Anger Happiness:0.0029 Sadness:0.0003 Surprise:0.0078 Neutral:0.089
17 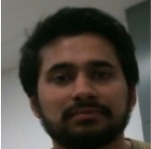 Happiness: 0.004723 Sadness: 0.02179761 Surprise: 0.01943829 Anger: 0.0009763971 Neutral: 0.9425282	18 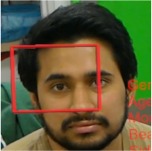 Gender: Male Age: 36.6 Emotion: Neutral Happiness:0 Sadness:0.01 Surprise:0 Anger:0	19 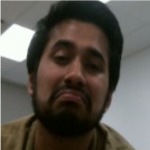 Happiness: 0. 1475 Sadness: 0.517531 Surprise: 0.0072156 Anger. 0.002564367 Neutral: 0.07885224	20 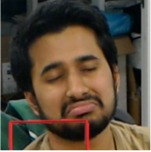 Gender: Male Age: 34.9 Emotion: Sadness Happiness:0.097 Surprise:0.048 Neutral:0.069 Anger:0.006
21 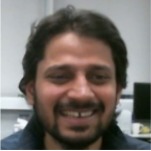 Happiness: 0.99999 Sadness: 3.3311x10${}^{-9}$ Surprise: 1.3208x10${}^{-9}$ Anger: 3.250402x10${}^{-9}$ Neutral: 6.1264x10${}^{-8}$	22 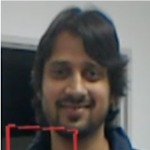 Gender: Male Age: 39.7 Emotion: Happiness Sadness:0 Surprise:0 Neutral:0 Anger:0	23 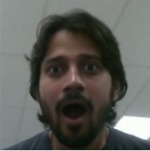 Happiness: 0.001138 Sadness: 7.764x10${}^{-5}$ Surprise: 0.9902573 Anger: 0.0006969759 Neutral: 0.004824177	24 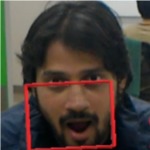 Gender: Male Age: 32.9 Emotion: Surprise Happiness:0.005 Sadness:0 Anger:0.001 Neutral:0.004
25 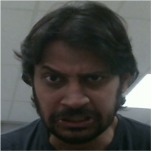 Happiness: 4.09x10${}^{-5}$ Sadness: 0.00691 Surprise: 0.005218 Anger: 0.6242462 Neutral: 0.3544377	26 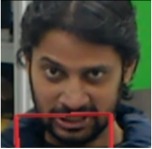 Gender: Male Age: 37.6 Emotion: Anger Happiness:0.00001 Sadness:0.00539 Surprise:0.09 Neutral:0.0156	27 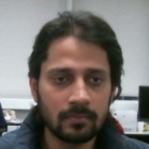 Happiness: 1.78x10${}^{-6}$ Sadness: 0.00046483 Surprise: 3.2659x10${}^{-6}$ Anger. 1.786311606 Neutral: 0.999515	28 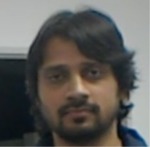 Gender: Male Age: 37.7 Emotion: Neutral Happiness:0.01 Sadness:0 Surprise:0 Anger:0
29 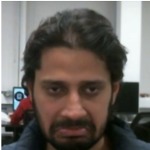 Happiness: 0.000573 Sadness: 0.5683 Surprise: 0.00052079 Anger: 0.00104 Neutral: 0.4325396	30 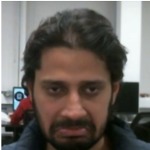 Gender: Male Age: 33.7 Emotion: Sadness Happiness:0.00001 Anger:0.0015 Surprise:0.0084 Neutral:0.21		
